# Piloting a blended-learning concept for integrating evidence-based medicine into the general practice clerkship

**DOI:** 10.3205/zma001279

**Published:** 2019-11-15

**Authors:** Bettina Engel, Miriam Esser, Markus Bleckwenn

**Affiliations:** 1 Carl von Ossietzky Universität Oldenburg, Department für Versorgungsforschung, Abteilung Allgemeinmedizin, Oldenburg, Germany; 2 Rheinische Friedrich Wilhelm University, Bonn, Germany; 3 University Bonn, Medical Faculty, Institut für Hausarztmedizin, Bonn, Germany

**Keywords:** evidence based medicine, blended learning, medical education, general practice clerkship

## Abstract

**Objective: **The present study investigates the feasibility of the application of evidence-based medicine (EBM) procedures by students as part of practical training in general medicine through a newly developed blended-learning teaching concept.

**Methodology: **This study describes the development, piloting and evaluation of a blended-learning concept for implementing EBM education as part of general practice training. Our concept consists of an online tutorial introducing the theoretical background, two classroom seminars for consolidation and practical exercises based on case studies. Following this, students were to apply their knowledge to real-life cases during their training. To evaluate the learning outcome, we have developed an evaluation tool based on the Fresno Test (Bonn Test). At the end of the seminar, students were invited to evaluate the concept.

**Results: **A total of 35 students took part in the feasibility study and 27 Bonn tests were evaluated. All students achieved more than the pass mark required in the Bonn Test in the preparation of clinical case studies. Because of the differentiated assessment of learning outcomes in all categories of the 5A EBM process, difficulties in the translation process can be revealed by the Bonn test. As a result, the concept can be refined and improved continuously. In the evaluation, 74% of the students rated the teaching concept "good" or "very good".

**Conclusion: **Overall, this study confirmed the feasibility of our EBM concept while demonstrating that students are able to apply the theoretical knowledge acquired to real-life settings. Further research with our concept is needed, especially at an earlier stage in the curriculum.

## Zusammenfassung

**Zielsetzung: **Die vorliegende Arbeit prüft die Machbarkeit der praktischen Anwendung von Evidenzbasierter Medizin (EbM) durch Studenten während des Blockpraktikums (BP) im Fachgebiet Allgemeinmedizin durch ein neu entwickeltes Blended-Learning-Lehrkonzept.

**Methodik:** Entwicklung, Pilotierung und Evaluation eines Blended-Learning Konzepts zur Einführung der Lehre der EbM im BP der Allgemeinmedizin werden beschrieben. Das Konzept besteht aus einem Online-Modul zur Vermittlung der theoretischen Grundlagen der EbM anhand des 5A-Schritteprozesses, zwei Präsenzseminaren zur Vertiefung der Inhalte und praktischen Übungen in Form von Fallbeispielen. Anschließend sollen die Studenten ihr erworbenes Wissen auf realen Patientenfällen in der Praxiszeit ihres BP anwenden. Zur Überprüfung des Lernerfolgs wurde auf Grundlage des Fresno Tests ein EbM-Evaluationsinstrument entwickelt – der Bonn- Test. Am Ende des Seminars können die Studenten das Konzept in einer schriftlichen Evaluation bewerten und Verbesserungsvorschläge einbringen.

**Ergebnisse: **Insgesamt nahmen 35 Studenten an der Machbarkeitsstudie teil, 27 Bonn-Tests konnten ausgewertet werden. Alle Studierende erreichten bei der Ausarbeitung klinischer Fallbeispiele mehr als die zum Bestehen benötigte Mindestpunktzahl im Bonn Test. Die differenzierte Leistungsdarstellung in den einzelnen Kategorien des 5-A-Schritteprozesses der EbM mit Hilfe des Bonn-Tests ermöglicht spezifische Schwierigkeiten bei der Translation von EbM-Wissen in die Praxis aufzudecken und das Lehrkonzept fortlaufend weiterzuentwickeln und zu verbessern. In der Evaluation bewerteten die Studenten das Lehrkonzept zu 74 % mit „gut“ oder „sehr gut“.

**Schlussfolgerungen:** Insgesamt bestätigt die vorliegende Studie, dass die Erweiterung des Blockpraktikums im Fachbereich für Allgemeinmedizin um das neu entwickelte EbM-Modul machbar ist und die Studenten die erlernten Inhalte erfolgreich in der Praxis anwenden können. Weitere Untersuchungen des Lehrkonzepts, insbesondere zu einem früheren Zeitpunkt des Medizinstudiums, sind empfehlenswert.

## Introduction

New scientific approaches and methods revolutionize the state of the art almost on a daily basis. Existing guidelines are constantly adapted to implement those scientific achievements in the clinical daily routine in a timely manner. In a meta review, Franke et al. showed that such innovations take a long time to get implemented in practice, if at all [1].

In order to treat the patients as required, the doctor in charge should be able to evaluate the relevant scientific literature [2]. Evidence-based medicine (EBM) provides doctors with the skills both for making balanced medical decisions and for lifelong learning. In order to familiarise future doctors with this area of responsibility at an early stage, EBM should become an integral part of the medical curriculum [2], [3]. Earlier studies clearly stated that the students are able to work on research questions during training in general practice, but they have to be well prepared and trained for this. Medical schools worldwide have already successfully integrated EBM into their curricula. Studies have shown that the integration of a blended-learning EBM unit in the medical curriculum or internship could close the gap between theory and practice [4], [5]. Unfortunately, this has not yet been incorporated into the licensing regulations in Germany, and not all medical schools have integrated EBM into their curricula. Even in Bonn, EBM is not yet an integral part of the curriculum. 

The focus of previous teaching approaches is often on the theoretical understanding of EBM. Dirk Mooshammer et al. published a contribution to the integration of scientific questions in the practical training in general medicine in year 5 [6]. In their general practical training, the students are already in an advanced stage of their medical education and therefore probably better able to formulate their own research questions in everyday practice. The present study examines the feasibility of evidence-based medicine practice by students during the training in general medicine as part of a newly developed blended learning teaching concept.

## Project description

The NKLM (National competence-based catalogue of learning objectives in medicine) not only requires students to understand the basics of EBM, but also to apply them in a problem-based manner [https://medizinische-fakultaeten.de/]. In order not only to train the students' theoretical understanding of EBM, but also to review translation in their clinical work, a new blended learning concept was developed in a dissertation. Prior to this, conceptual criteria were defined in order to be able to evaluate the practical implementation of EBM:

1. Learning Outcomes: Students should have sufficient basic education in the field of EBM to allow them to understand the concept of evidence-based medicine. In particular, knowledge management should be trained here.
2. Universal validity: The content of learning should be generalizable and thus applicable to a wide range of issues in everyday clinical practice.
3. Evaluability: In order to be able to evaluate the developed teaching concept or the acquired competences, evaluation instruments have to be designed.
4. Compliance: The teaching concept can only be successfully implemented if the perspective of the students is also included in the development of the concept.

Based on these 4 criteria, a multi-level teaching concept has been developed. This includes a blended-learning online module, two on-site seminars, worksheets, and a procedure to review students' practical EBM skills and finally an evaluation tool. In terms of time, the sequence was as shown in table 1 (Tab. 1).

### Participants

We chose three different trainee groups in different time slots in the academic year as a participating group. Each group had between 10 and 15 participants. Participation in the EBM concept was formulated as a voluntary additional offer.

Of the total of 38 students in the three participating groups, a total of 35 attended the first classroom seminar.

#### On-line tutorial

After a training course held at the University Computing Center Bonn, ME developed a module for the eCampus online platform, which is used by numerous institutes to provide teaching material to students. A pre-test with four students further improved the user-friendliness of the online application. The content of the learning module is divided into a general introduction to evidence-based medicine and chapters on each sub-item of the 5-A step process. The 5-A-step process is the theoretical basis of evidence-based medical practice [3], [7]. It should be understood as a cycle of processes. The 5 As stand for Ask (formulation of the question), Acquire (knowledge acquisition), Appraise (evaluation of findings), Apply (application of knowledge) and Assess (evaluation of effects).

Likewise, the PICO scheme is introduced and explained. The PICO scheme is used to develop scientific questions [8]. The letters P, I, C and O stand for the most important aspects that should be considered if searching for the best possible therapy (P=“Patient Problem”, I=“Intervention”, C=“Comparison”, O=“outcome”). In order to make self-study with the learning module as interactive as possible, three to four repetition questions are interposed between the individual chapters. Once the students have completed a chapter, they can use the multiple choice and assignment questions to immediately review their understanding of the key content. An interactive case study helps to go through the individual steps again in order to eliminate possible questions before processing the work assignment.

#### Home work

Following the e-learning module, the trainees were to apply the 5-A step process in practice. For this purpose, 3 case studies have been developed. The students were assigned to one of the three cases beforehand by e-mail. Based on 10 given questions (BONN Test), students were to apply their basic knowledge.

#### Selection of case studies

The three case studies A, B and C are all related to gout research. This topic was chosen by BE because of her expertise in this field. She is one of the authors of the guideline for acute and chronic gout of the German College of General Practitioners and Family Physicians.

Development of an EBM evaluation tool

The Fresno Test and the Berlin Questionnaire [9], [10], [11] serve as validated tools for reviewing skills in evidence-based medicine. The "Bonn Test" was developed on the basis of the Fresno test (see table 2 (Tab. 2)). In the original Fresno test, the first 4 As and additional basic statistical knowledge are queried. The Bonn Test covers all 5 As and consists of 10 open questions, allowing the test to be applied to any case study. Since the learning goals of our module did not include any statistical or mathematical knowledge, we have excluded these fields for the benefit of constructive alignment.

For each question, an expectation horizon has been set (see table 3 (Tab. 3)) which defines the content that needs to be covered in the answer, as well as the level of detail, to achieve a certain score.

## The classroom seminars

Every Monday, Students take part in a 3-hour seminar in the Institute of Family Medicine in Bonn during their two-week training in general practice. The aim of the seminar is to deepen the contents of the online learning module as well as to discuss students’ homework. The seminars were led by a general practitioner and research associate at the Institute of Family Medicine (BE) and the PhD student (ME).

### The first classroom seminar

The first seminar begins with a short introductory round in which the students are to describe their level of satisfaction with the e-learning module, any comprehension issues, as well as expectations and wishes regarding their clinical training in general practice. Next, the lecturer makes a compact PowerPoint presentation on EBM. This is to refresh the knowledge acquired and is not intended to replace the self-study with the e-learning module. This is followed by an interactive unit where students are asked to assign cards with different study types to the correct definitions on a bulletin board and arrange them according to their levels of evidence. Thereafter, the students discuss different sources of research with the lecturer. Although we chose PubMed as the medium for our e-learning module because it helps us to follow each step, students should also familiarise themselves with commonly used sources such as the Cochrane Library or UptoDate. Finally, the students present their own search strategies and research results of their homework. They first compare their findings in small groups with students who have worked on the same case. Each group then presents a completed Bonn Test. The findings presented are then discussed together in the large group.

#### Work assignment

At the end of the first seminar, the students receive a work assignment for the training in general practice. Until the next seminar on the following Monday, they should develop their own research questions during their stay in the teaching practices and work on them with the help of the Bonn Test. The students are free to choose the topic. The students are expected to bring the completed documents with them for the second seminar.

#### The second classroom seminar

During the second seminar, the students can exchange their experiences in the training in general practices while also addressing any problems and ambiguities. The students present their self-developed research questions on clinical cases from the training as well as their research results and therapy recommendations. Afterwards, one of the questions presented will be resolved online so students can experience the practical application of EBM. At the end of the seminar, the completed documents for the students’ own clinical questions will be collected. They can be evaluated with the help of the expectation horizon of the Bonn Test, in much the same way as the gout case studies. The performance of the students is assessed in relation to the peer group and by comparing the theoretical with the practical application.

#### Evaluation

Evaluation sheets were handed out at the end of the second seminar. This evaluation includes the satisfaction of the students with the online tutorial, the seminars as well as the training in the practices. Criteria covered by the evaluation include the didactic and technical implementation of the concept as well as the learning effect for the respective aspects of the teaching concept.

## Results

Through our feasibility study, a total of three data sets could be generated.

**Figure 1:**
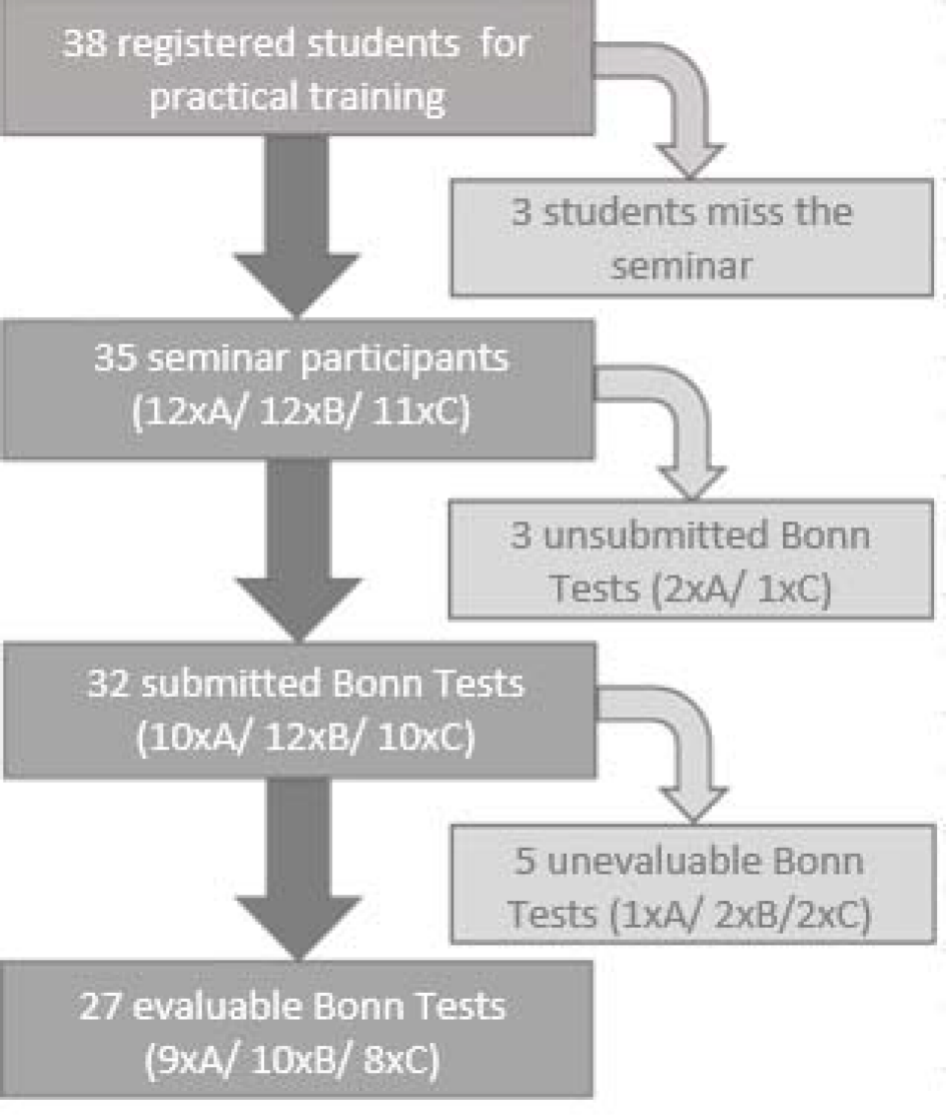
Recruitment of evaluable Bonn tests

**Figure 2:**
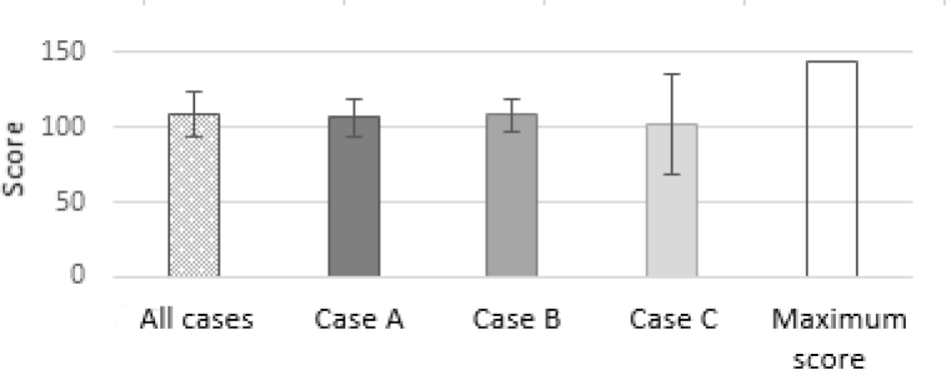
Presentation of the achieved score in the Bonn Test for the notional cases and direct comparison of all contributions with the case-specific contributions A, B and C as well as the maximum score (Max 144) (see table 3).

**Figure 3:**
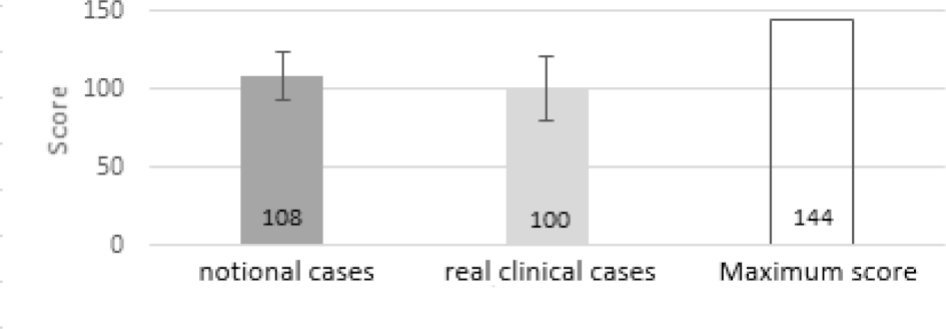
Comparison of the average score for notional cases and real clinical cases

**Figure 4:**
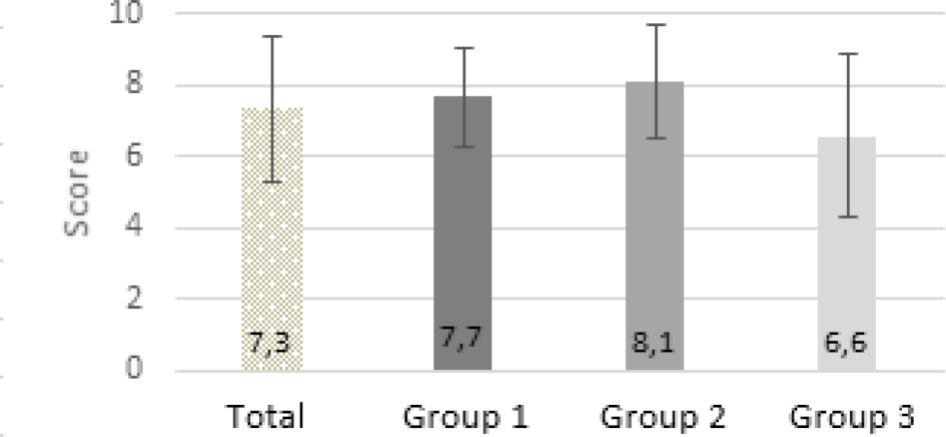
Presentation of the evaluation results for the EbM concept (maximum score: 10)

The result of the Bonn Test is available for the gout case studies A, B and C. With this data, the general applicability of the teaching concept under controlled conditions is examined. In total, 32 of the 35 seminar participants (91%) submitted their gout case study (see figure 1 (Fig. 1)). All of the 27 evaluable contributions achieve at least the required half of the possible maximum score (maximum 144 points). On average, 108.4 points are achieved with a standard deviation of 15.4 points. In the direct comparison, the students in the case studies A (MW106.2, SD=12.7, n=9), B (MW=107.9, SD=10.9, n=10) and C (MW=111), 6; SD=21.3; n=8) are similarly good, but case C shows a higher deviation from the mean result (see figure 2 (Fig. 2)).
2. The self-developed research questions of the students are evaluated with the Bonn Test and the quality of the solution approaches is evaluated according to the horizon of expectation. These data provide an insight into the translation of the learned contents into practice. The differentiated presentation of performance in all categories of the 5-A-step process with the aid of the defined points in the expectation horizon allows the direct comparison with the theoretical application of EBM. On average, the total score of the evaluated tests is 99.8 points (s=20.5) below the mean for the notional cases A, B and C (MW=108.4, SD=15.4) (see figure 3 (Fig. 3)).
3. The third data set is the evaluation questionnaire designed to assess students' satisfaction with the extension of the traineeship as well as their self-assessment of newly acquired skills. The most important aspects of the teaching concept are evaluated using a numerical scale from 0 (“does not apply”) to 10 (“fully agrees”). Out of a total of 34 evaluation forms, 31 were completely filled out and were considered in the further procedure. Overall, 74% of students rated the extension of the traineeship as good or very good (see figure 4 (Fig. 4)). In group one and two, it is even 83% and 82% respectively. On average, the third group rates the change worse than the block trainees of the first two test runs with 6.6 out of 10 points (s=2.3). All of the trainees are in favour of implementing the teaching concept at an earlier stage in the curriculum.

## Discussion

We were able to show that our teaching and learning concept, in the sense of “constructive alignment”, combines students' self-directed learning with the assignment of relevant tasks [12]. On the basis of relevant and realistic tasks, the students were able to achieve the intended learning objectives (fundamentals of scientific work). The results of the Bonn Test for the notional case studies A, B and C suggest that the students reached the desired learning goals in the field of EBM at the end of the learning module. The expectation horizon of the Bonn Test can specifically capture the newly acquired skills and appropriately differentiate strengths and weaknesses in relation to the 5-A-step process. Even if the average score of the Bonn Test is in practice below the score for the application in theory, it still by far exceeds the minimum score required to pass. This shows that both the content of the learning module as well as the assigned tasks can be transferred from theory into practice. The lower average score for “real cases” in some categories is probably due to the complexity of patient cases, which requires even more practice to systematically apply the 5-A step process. The self-developed research questions can also be processed and evaluated with the Bonn Test. This shows the translation of the theoretical contents into practice.

Although Ilic et al. showed no superiority of blended learning over classroom education, the group has been able to demonstrate that a combination of both methods results in a significantly better translation of EBM into practice [13]. The expectation horizon of the Bonn Test is an adequate evaluation tool for the relevant EBM skills with real patient cases just as with the notional case studies.

Overall, we found potential for improving the concept.

Particularly in the development of adequate questions or search strategies, the students need further support.

The Bonn Test is associated with a labor-intensive and time-consuming correction process due to its open formulations and its corresponding horizon of expectation. This makes implementation difficult for all students at the same time. On the other hand, the open formulations are the greatest strength of the Bonn Test, as they allow the evaluation of the practical application of EBM at this depth. Therefore, a validation of the Bonn Test, if necessary in abbreviated form, appears as a useful further research approach.

The evaluation shows a high acceptance for the extension of the traineeship, so that a future implementation of the teaching concept into the curriculum is feasible. However, the students unanimously agree that it would make more sense to implement this at an earlier phase in their studies. The evaluation reveals that the closeness to the state exam is highly relevant: the later the time of the participating group was in the academic year, the more the students saw the extra workload as a problem due to the upcoming examinations.

In the future implementation of the blended learning module, external framework conditions must be taken into account to a higher degree. The implementation of the EBM module during the traineeship, i.e. in the 5th year, does not appear to be appropriate according to the evaluation results. Instead, the EBM module could provide students with the basic skills right at the beginning of the clinical phase in year 3. In the subsequent cross-sectional phases, the students would then be able to work on increasingly complex research questions of their own. The implementation of a correspondingly revised longitudinal model of our EBM module is currently being planned.

Also for cost reasons, the integration of a blended learning module as a possible teaching method is a reasonable alternative. Maloney et al. were able to show that the development and implementation costs of a blended learning unit achieve the break-even point after three years compared to classroom training [14].

Moreover, this approach also fits in with the requirements of the Master Plan for Medical Studies 2020, which postulates that more scientific competences should be taught throughout the course of study.

## Conclusion

Our results show that the developed EBM concept is a promising model for the implementation of evidence-based medicine in the curriculum. It enables medical students for the use of EBM in everyday practice. Overall, the present study confirms that the extension of the curriculum in the field of general medicine is feasible for EBM training and that students can successfully apply the learned content in practice. With the help of the evaluated Bonn Tests and student feedback, the teaching concept can be further improved. The Bonn Test seems to be an adequate evaluation tool for the practical application of EBM. Additional studies, especially at an earlier stage during medical studies, should further examine the validity of the Bonn Test.

## Competing interests

The authors declare that they have no competing interests. 

## Figures and Tables

**Table 1:**
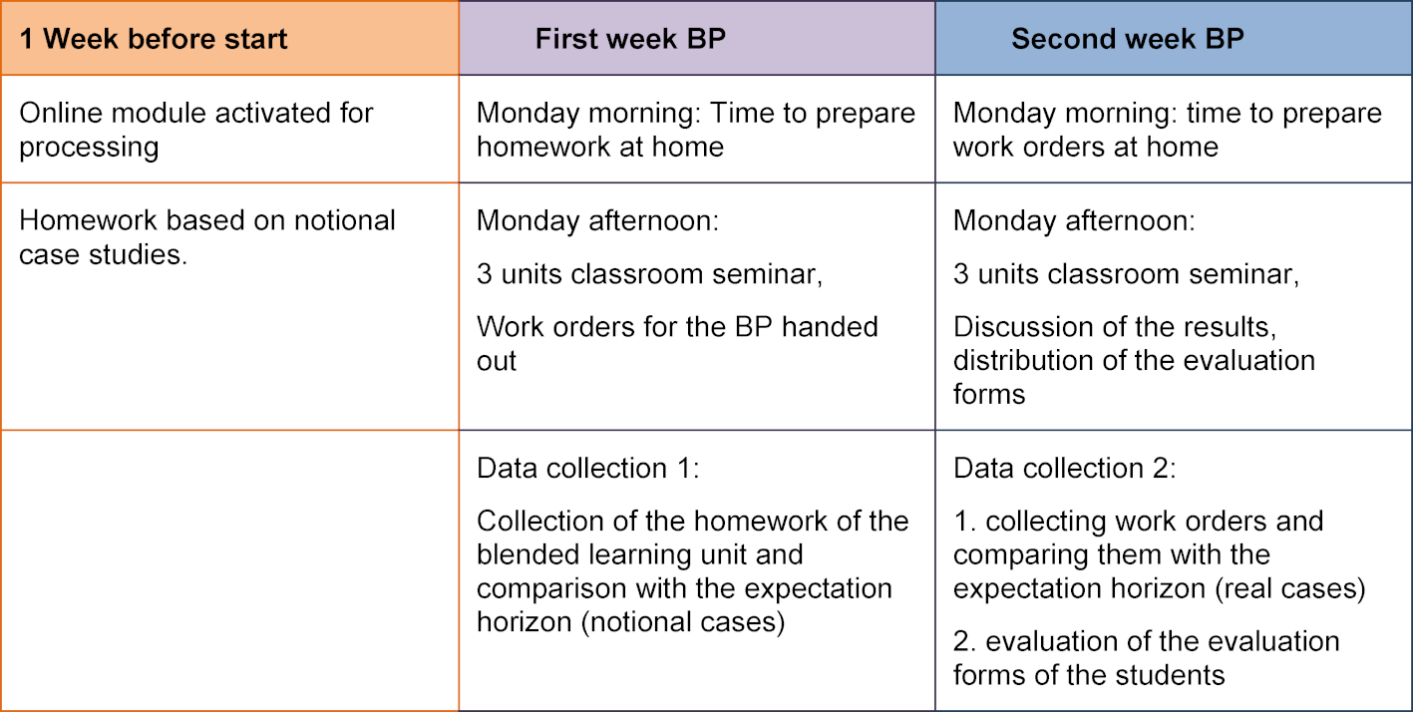
The chronological order of the EbM teaching concept as part of a two-week block clerkship (BP) in General Medicine

**Table 2:**
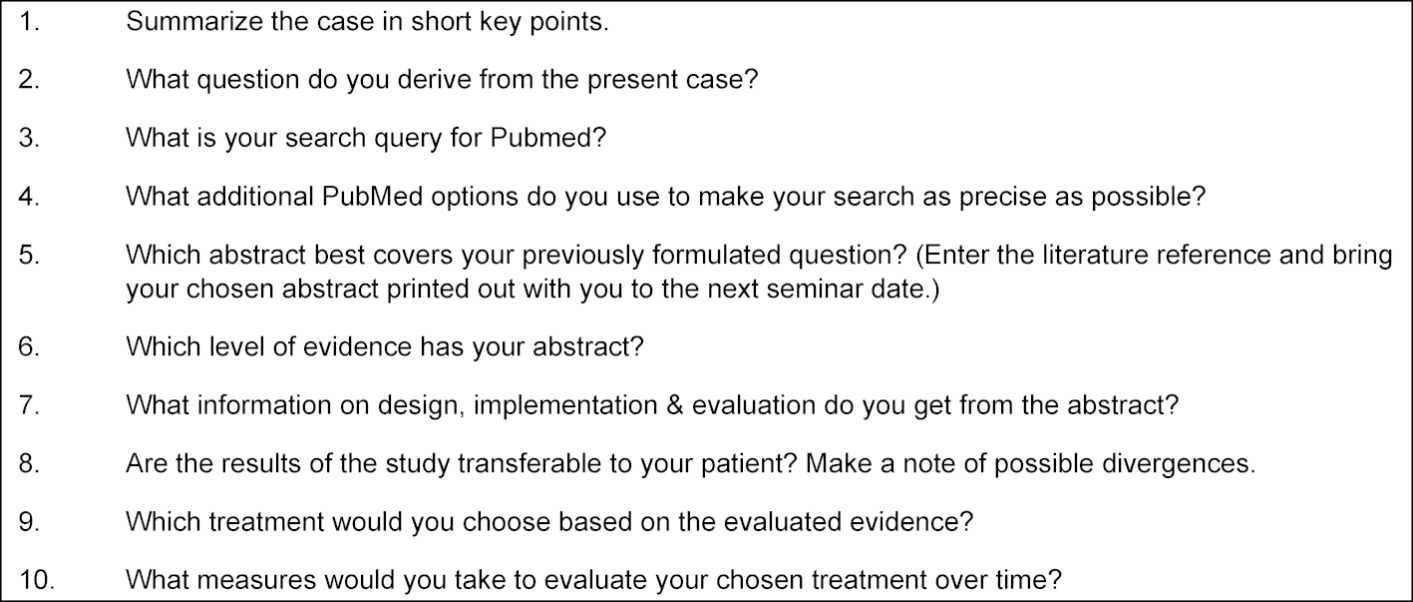
Shown are the questions of the Bonn Test. For the evaluation of the Bonn Test, a corresponding expectation horizon with four-step grading was created (see table 3).

**Table 3:**
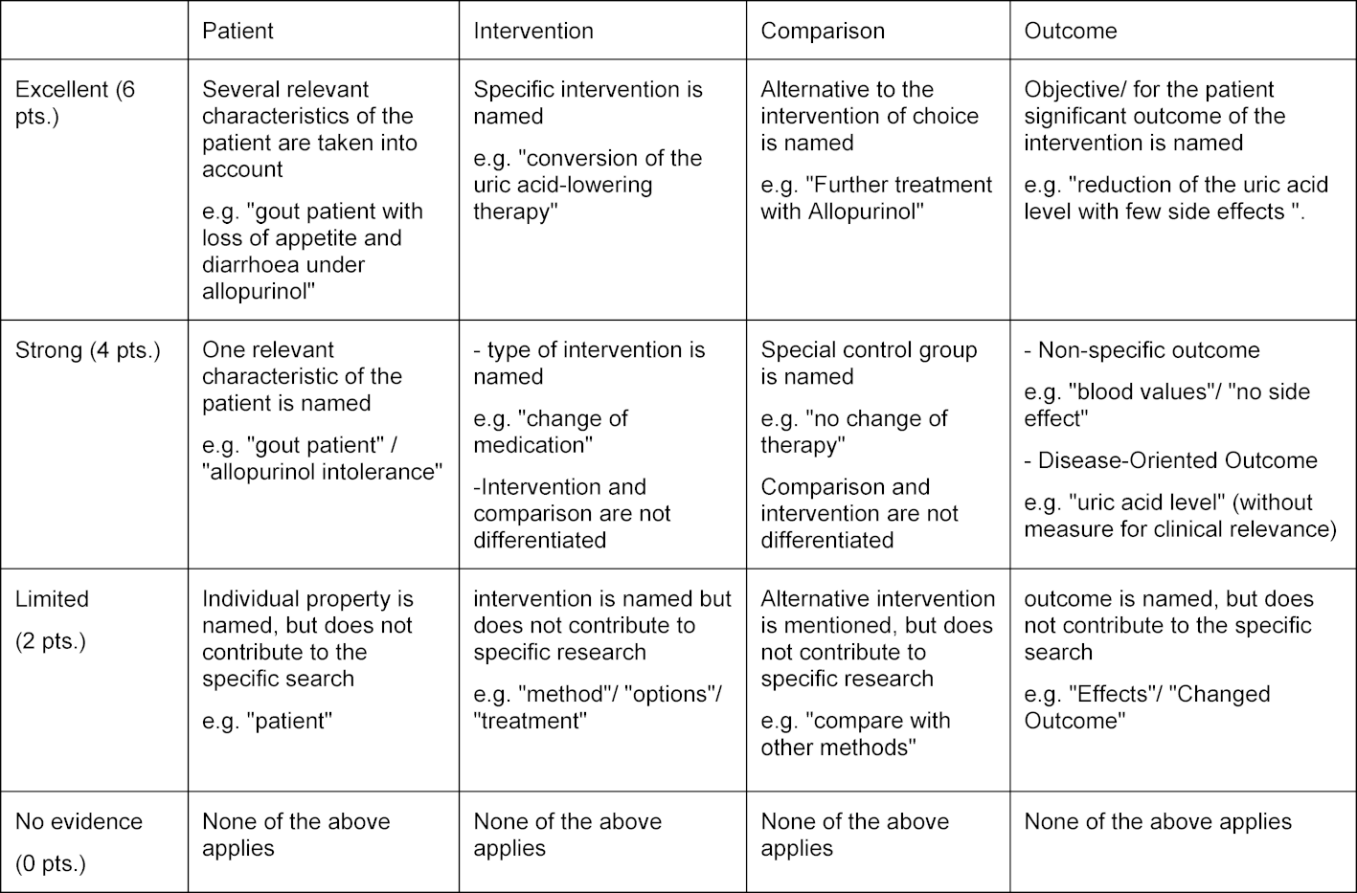
Extract from the Bonn Test (expectation horizon of the step “Ask”)
